# Hydrophilization of Poly(Caprolactone) Copolymers through Introduction of Oligo(Ethylene Glycol) Moieties

**DOI:** 10.1371/journal.pone.0099157

**Published:** 2014-06-16

**Authors:** Jonathan J. Wurth, Nils R. Blumenthal, V. Prasad Shastri

**Affiliations:** 1 Institute for Macromolecular Chemistry, University of Freiburg, Freiburg, Germany; 2 BIOSS Centre for Biological Signaling Studies, University of Freiburg, Freiburg, Germany; Instituto de Engenharia Biomédica, University of Porto, Portugal

## Abstract

In this study, a new family of poly(ε-caprolactone) (PCL) copolymers that bear oligo(ethylene glycol) (OEG) moieties is described. The synthesis of three different oligo(ethylene glycol) functionalized epoxide monomers derived from 2-methyl-4-pentenoic acid, and their copolymerization with ε-caprolactone (CL) to poly(CL-*co*-OEG-MPO) copolymers is presented. The statistical copolymerization initiated with SnOct_2_/BnOH yielded the copolymers with varying OEG content and composition. The linear relationship between feed ratio and incorporation of the OEG co-monomer enables control over backbone functional group density. The introduction of OEG moieties influenced both the thermal and the hydrophilic characteristics of the copolymers. Both increasing OEG length and backbone content resulted in a decrease in static water contact angle. The introduction of OEG side chains in the PCL copolymers had no adverse influence on MC-3TE3-E1 cell interaction. However, changes to cell form factor (Φ) were observed. While unmodified PCL promoted elongated (anisotropic) morphologies (Φ = 0.094), PCL copolymer with tri-ethylene glycol side chains at or above seven percent backbone incorporation induced more isotropic cell morphologies (Φ = 0.184) similar to those observed on glass controls (Φ = 0.151).

## Introduction

Biodegradable polyesters are widely used in various biological and medical applications [1]–[3] such as tissue engineering [4], [5], injectable and implantable drug delivery systems [6], medical devices, orthopaedic implants [7], and bioactive coatings [8]. In addition, degradable polyesters derived in particular from lactic acid have found a niche as a renewable resource for ecofriendly packaging [9], [10].

Poly(ε-caprolactone) (PCL) is a semi-crystalline degradable polyester with a good physicochemical profile that make it suitable for many biomedical applications [1], [6], [11], [12]. The melting temperature (T_m_) which is in the range of 56°C to 65°C is well suited for melt processing and compounding with bioactives such a small molecule drugs and peptides. With a glass transition temperature (T_g_) around −56°C to −65°C [12], [13], PCL is a soft, pliable material at physiological conditions and is well suited for implantation. The high crystallinity of PCL up to 69% [13] in comparison to poly(glycolic acid) (PGA) (46% to 52%), poly(l-lactic acid) (l-PLA) (0% to 7%), and poly(d,l-lactic acid) (d,l-PLA) (amorphous) results in the formation of phase separated microstructures composed of large spherulites. This poses some challenges in achieving homogeneous degradation *in vivo*, but affords considerable life-time of 24 to 36 months under physiological conditions [14]. PCL in comparison to PLA or PGA is considerably more hydrophobic [15]. This decreased hydrophilicity derives from the polymer backbone that consists of hydrophobic aliphatic hexane units that are linked with ester moieties which limits water uptake and therefore prolongs the onset of hydrolytic degradation. As a result, the degradation lifetime of PCL is longer in comparison to PLA, PGA and its copolymers, thus diminishing its potential impact as a biomedical material. Additionally, in spite of eco-credentials of PCL it has seen limited use outside the medical arena and this could change if new strategies are developed to introduce diversity in chemical structure and function.

PCL is synthesized primarily by ring-opening polymerization (ROP) using stannous octaoate [12]. In this ROP approach, primary and secondary alcohols are routinely used as co-initiators and this has been leveraged to introduce elements along the PCL backbone. Thus far various routes and concepts have been established in the literature to improve the hydrophilicity of PCL [16]. Poly(ethylene glycol) (PEG) is commonly used because of its hydrophilicity, excellent biocompatibility and anti-fouling characteristics [17]–[21]. PEG incorporation into PCL backbone has been achieved using several approaches to form di- and tri-block copolymers, end-capped copolymers, graft-copolymers, and statistical copolymers [22]–[26]. In particular, Cho and Park [23] have presented an approach to synthesize a statistical copolymer composed of CL and epichlorohydrin functionalized PEG [17]–[19], [22]–[26]. We recently described the synthesis of functionalized PCL via the copolymerization of ε-caprolactone (CL) with a novel α,ω-epoxy esters derived from 2-methyl-4-pentenoic acid [27]. In that study we demonstrated that ethyl 2-methyl-4-pentenoateoxide could be copolymerized with CL using a SnOct_2_/BnOH initiating system. Furthermore, we showed that the introduced functionalities are discretely distributed along the polymer backbone resulting in the statistical copolymers of well defined backbone architecture [27], [28]. These functionalized epoxides therefore combine the advantages of a predictable copolymerization system with known reaction parameters, with a versatile system that allows a wide range of functional moieties in the ω-position of the epoxide.

**Figure 1:**
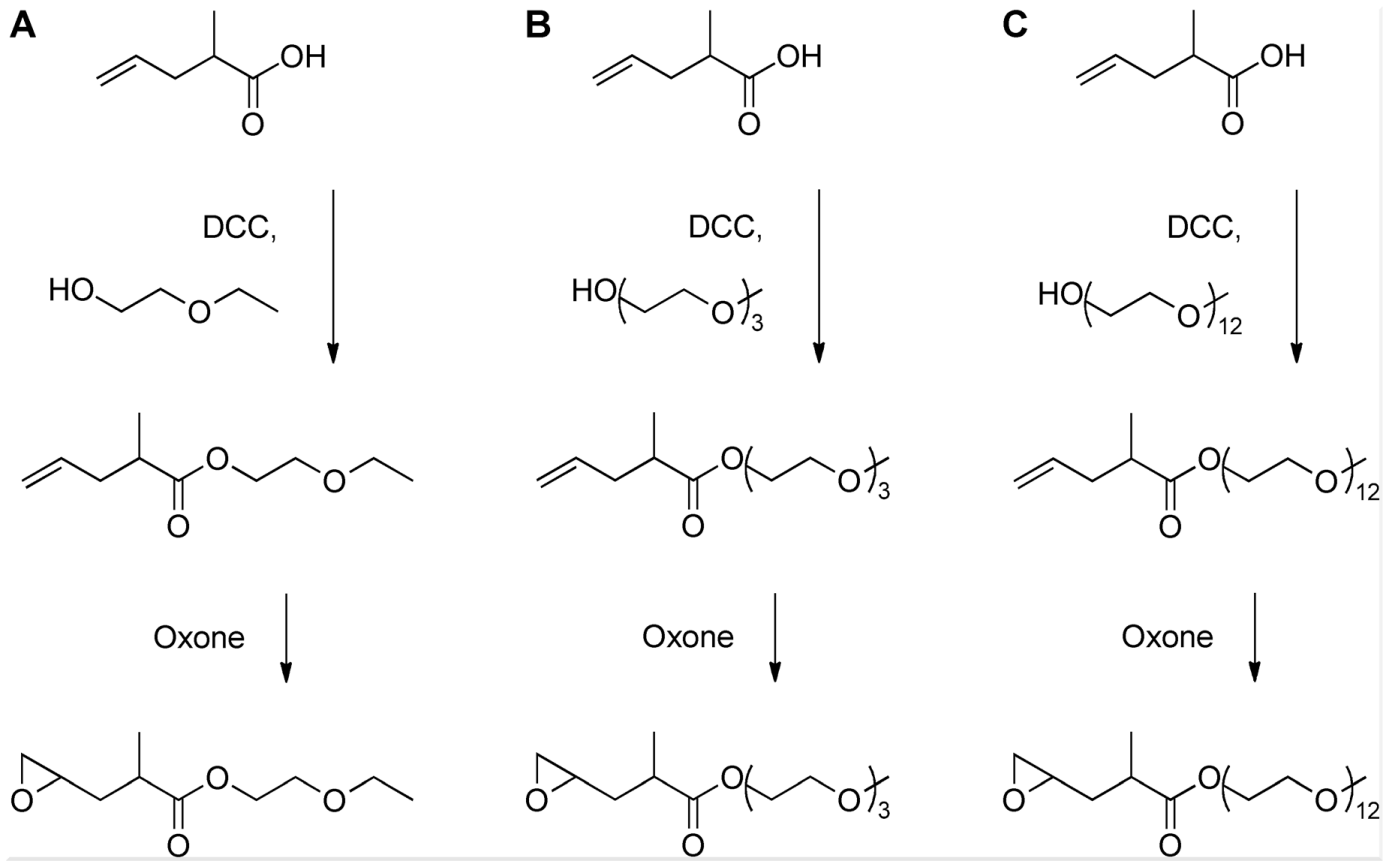
Synthesis of OEG functionalized epoxides.

**Figure 2:**
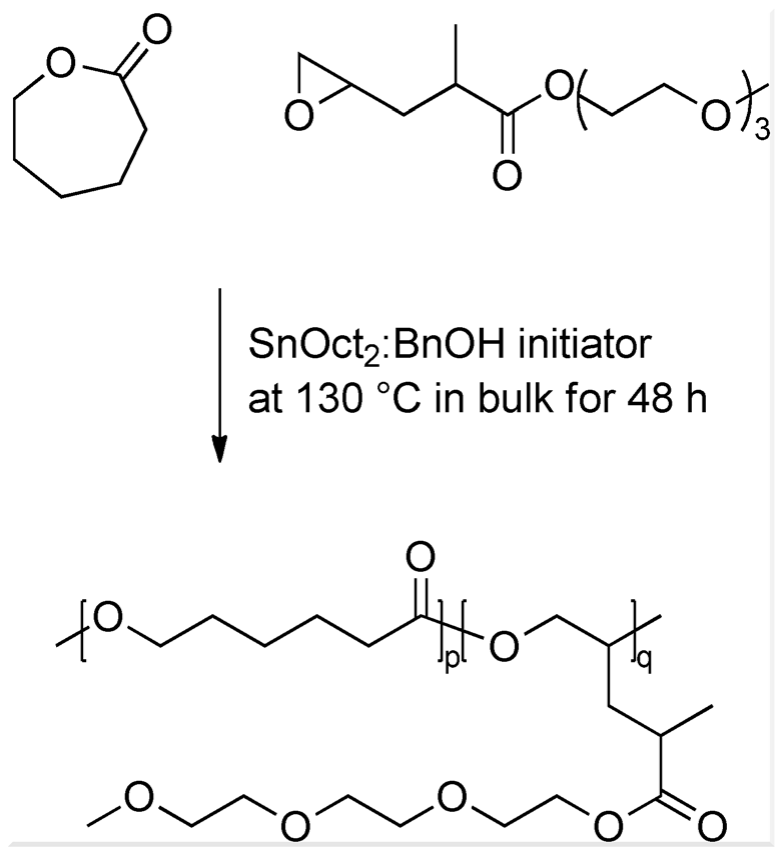
^1^H-NMR spectra of OEG-MPO epoxides. A,1EG-MPO; B, 3EG-MPO; C, 12EG-MPO.

In this work we build on the successful functionalization of PCL using α,ω-functionalized epoxides and present a strategy for introducing hydrophilic oligo(ethylene glycol) (OEG) moieties into the PCL backbone using OEG-ω-epoxides. In order to establish a structure-property-function relationship for hydrophilization, a series of epoxy esters bearing ethylene glycol (EG) oligomers of different chain length were synthesized through the esterification of methyl-pentenoic acid (MPA) followed by Oxone oxidation to yield OEG esters of methyl-2-pentenoate oxide (OEG-MPO) (**Figure 1**). The synthesis of poly(CL-*co*-OEG-MPO) copolymers was carried out using standard Tin(II) catalyst (**Figure 2**). The bulk and surface properties of the copolymers were characterization and interaction with murine pre-osteoblasts (MC3T3-E1) with spun-cast copolymers films was evaluated.

## Materials and Methods

### Materials

All chemicals *N*,*N*′-Dicyclohexylcarbodiimide (DCC), 2-ethoxyethanol (1EG), triethylene glycol monomethyl ether (3EG), poly(ethylene glycol) monomethyl ether (12EG, M = 550 g/mol), 2-methyl-4-pentenoic acid (MPA), and 4-(dimethylamino)pyridine (DMAP), dichloromethane (DCM), tetrahydrofuran (THF), were purchased from Sigma-Aldrich and used as received unless stated otherwise. Sodium bicarbonate solution and sodium phosphate buffer solution (PBS, 1×) were prepared in house.

### Monomer synthesis

The functionalized epoxides 2-ethoxyethanol 2-methyl-4-pentenoate oxide (1EG-MPO), triethylene glycol monomethyl ether-2-methyl-4-pentenoate oxide (3EG-MPO), and poly(ethylene glycol) monomethyl ether-2-methyl-4-pentenoate oxide (12EG-MPO) were synthesized via a two-step synthesis involving esterification followed by epoxidation [28].

For the synthesis of OEG ester, a one liter round bottomed flask was charged with *N*,*N*′-dicyclohexylcarbodiimide (36.11 g, 1.0 eq, 175 mmol) dissolved in 300 ml DCM and cooled to 5°C using an ice bath, following which the alcohol (2-ethoxyethanol (18.02 g, 1.3 eq, 200 mmol), or triethylene glycol monomethyl ether (32.84 g, 1.3 eq, 200 mmol), or poly(ethylene glycol) monomethyl ether (M = 550 g/mol, 110.00 g, 1.3 eq, 200 mmol)) was added as a solution in DCM (50 ml). Following this, 2-methyl-4-pentenoic acid (MPA) (24.89 g, 1.0 eq, 175 mmol) dissolved in 50 ml DCM and a catalytic amount of 4-dimethylamino pyridine (1 g) were added and the mixture was stirred overnight with gradual warming to room temperature. After 18 hours, the reaction mixture was filtered off and the filtrate was washed with aqueous sodium bicarbonate solution (half saturated, 600 ml) by stirring for 2 hours. The mixture was extracted with DCM (8×25 ml), the combined organic layers were dried over sodium sulfate and the solvent was removed to obtain the intermediate product.

Epoxidation was carried out using a procedure described recently [27]. The OEG ester of MPA (OEG-MP), (1.0 eq, 150 mmol) was dissolved in a mixture of acetone (300 ml) and sodium phosphate buffer solution (300 ml, c = 0.2 mol/l, pH = 7.0) in a 2-liter three-necked round bottomed flask quipped with a dropping funnel. The reaction mixture was cooled down to 5°C using an ice bath and the pH was set to 7.2 using sodium hydroxide solution (c = 2 mol/l). A solution of the persulfate salt Oxone (92.21 g, 2.0 eq, 300 mmol) in water (400 ml) was added dropwise over 2 h. During addition, the pH was kept between 7.2 and 7.5 using NaOH solution (c = 2 mol/l) and the temperature was kept below 5°C. After addition, the pH was controlled for another 2 h and the reaction mixture was kept stirring overnight. After 18 hours, the white precipitate was filtered off and the reaction mixture was extracted with DCM (8×25 ml). The combined organic layers were dried over sodium sulfate and the solvent was removed. The crude product was purified by flash column chromatography (silica, *i*-hexane–ethyl acetate eluent) to yield the pure epoxide of the OEG-MP (OEG-MPO).

#### 1EG-MPO

C_10_H_18_O_4_, M_1EG-MPO_ = 202.25 g/mol. ^1^H-NMR (300 MHz, CDCl_3_): δ (ppm) = 1.1–1.3 (m, 6H) 1.5–1.7 (m, 2H), 2.5 (m, 1H), 2.7 (m, 2H), 3.0 (m, 1H), 3.5 (q, 2H), 3.6 (t, 2H), 4.2 (m, 2H). EA: C 58.85% (58.39%), H 9.10% (8.97%).

#### 3EG-MPO

C_13_H_24_O_6_, M_3EG-MPO_ = 276.33 g/mol. ^1^H-NMR (300 MHz, CDCl_3_): δ (ppm) = 1.4 (m, 3H), 1.5–2.0 (m, 2H), 2.4 (m, 1H), 2.7 (m, 2H), 3.0 (m, 1H), 3.4 (s, 3H), 3.5–3.7 (m, 8H), 4.3 (t, 2H). EA: C 55.81% (56.51%), H 9.01% (8.75%).

#### 12EG-MPO

C_31_H_60_O_15_, M_12EG-MPO_ = 672.80 g/mol. ^1^H-NMR (300 MHz, CDCl_3_): δ (ppm) = 1.2–1.3 (m, 3H), 1.5–2.0 (m, 2H), 2.5 (m, 1H), 2.6–2.8 (m, 2H), 3.0 (m, 1H), 3.4 (s, 3H), 3.6 (m, 44H), 3.7 (m, 2H), 4.2 (m, 2H). EA: C 53.88% (55.34%), H 9.01% (8.99%).

### Copolymerization

The copolymerization was carried out as a 50-mmol batch. A 50 ml two-neck round bottomed flask was charged with ε-caprolactone (80%–100%, 40 mmol–50 mmol) and an OEG-MPO (1EG-MPO, 3EG-MPO, or 12EG-MPO, up to 20%, (10 mmol). DCM (20 ml) was added to ensure complete dissolution and mixing. The reaction mixture was heated to 130°C and the volatile DCM was evaporated. A SnOct_2_∶BnOH initiator solution in chloroform (0.2 mol%, 1 ml stock solution, c = 0.1 mmol/ml) was then added and the polymerization was run for 48 h at 130°C. The polymerization was quenched by adding a solution of dichloromethane and ethanol (2 ml, DCM∶EtOH 9∶1). The polymer was purified by solubilization in DCM followed by precipitation in cold ethanol and collection of the precipitate by vacuum filtration. The polymer was finally dried in vacuum over 48 h, at room temperature before further analysis.

#### Poly(CL-*co*-1EG-MPO)

^1^H-NMR (300 MHz, CDCl_3_): δ (ppm) = 1.2 (t, 3H_MPO_), 1.2–1.5 (m, 2H_CL_, 3H_1EG_), 1.5–1.7 (m, 4H_CL_, 2H_MPO_), 2.2–2.4 (m, 2H_CL_, 1H_MPO_), 3.5–3.7 (m, 2H_MPO_, 4H_1EG_), 4.0–4.1 (m, 2H_CL_), 4.1–4.3 (m, 1H_MPO_, 2H_1EG_).

#### Poly(CL-*co*-3EG-MPO)

^1^H-NMR (300 MHz, CDCl_3_): δ (ppm) = 1.1–1.3 (m, 3H_MPO_), 1.3–1.5 (m, 2H_CL_), 1.5–1.8 (m, 4H_CL_, 2H_MPO_), 2.2–2.4 (m, 2H_CL_, 1H_MPO_), 3.4 (s, 3H_3EG_), 3.5–3.7 (m, 2H_MPO_, 10H_3EG_), 4.0–4.1 (t, 2H_CL_), 4.2–4.3 (m, 1H_MPO_, 2H_1EG_).

#### Poly(CL-*co*-12EG-MPO)

^1^H-NMR (300 MHz, CDCl_3_): δ (ppm) = 1.1–1.3 (m, 3H_MPO_), 1.3–1.5 (m, 2H_CL_), 1.5–1.7 (m, 4H_CL_, 2H_MPO_), 2.2–2.4 (m, 2H_CL_, 1H_MPO_), 3.4 (s, 3H_12EG_), 3.5–3.8 (m, 44H_12EG_), 4.0–4.1 (t, 2H_CL_), 4.2–4.3 (m, 1H_MPO_, 2H_12EG_).

### Copolymer characterization

#### ^1^H-Nuclear magnetic resonance (NMR) spectroscopy

NMR spectroscopy measurements were carried using an ARX 300 MHz spectrometer (Bruker, USA). Samples (5 mg–50 mg) were dissolved in CDCl_3_ (0.7 ml) and measured at 25°C. The spectra were analyzed using Bruker Topspin 3.1 and chemical shifts were expressed in ppm with respect to the CHCl_3_ signal at 7.26 ppm [29].

#### Size exclusion chromatography (SEC)

SEC was carried out using 1200 Series GPC-SEC (Agilent Technologies, USA) that was calibrated against EasiVial PS-H standard. Polymers were dissolved in THF (c = 4 mg/ml) and measured with a flow rate of 1 ml/min. Moelcualr weight and polydispersity index (PDI) was determined using WinGPC Unity software (Polymer Standard Solutions, USA). The PDI, which is the ratio of the weight average molecular weight to number average, is an indicator of the homogeneity of the polymer chains. A PDI of ≤2 is indicative of a fairly homogeneous polymer population.

#### Thermal Analysis

Differential scanning calorimetry (DSC) was carried out using Pyris 1 calorimeter (Perkin-Elmer, USA) at a heating rate of 10°C/min. A known mass of polymer (5 mg–7 mg) was directly weighed in the DSC aluminum pans and sealed using a crimping device. As a pretreatment, the sample was heated to 150°C to remove thermal history and then cooled to −90°C. The sample was heated to 150°C with a heating rate of 10°C/min to determine the melting temperature (T_m_) and at a heating rate of 40°C/min to determine the glass transition temperature (T_g_).

#### Polymer film preparation for surface analysis

The polymer films for contact angle measurements, film roughness analysis and cell culture were spun-cast using a spincoater (Model P6700, Specialty Coating Systems, USA). Half a milliliter of polymer solution in THF (10 wt%) was dropped on a clean, dust free glass slide (d = 25 mm) and spin-cast using the following spinning regimen: 20 sec at 120 rpm and then 20 sec at 2000 rpm. The films were dried in vacuum at 25°C overnight to remove solvent residues.

#### Atomic force microscopy of copolymer films

The surface roughness of the copolymer films was determined using a Dimension V (Veeco Instruments, USA) atomic force microscope (AFM). Surface roughness is reported as root mean square (Rq) values which was determined using the inbuilt function in the NanoScope software (Version 1.20).

#### Water contact angle (CA) measurement

CA were measured using a goniometer system equipped with a digital camera for droplet image capture (CAM 2008, KSV Instruments, Finland) using the sessile drop method [30]. A drop of distilled water (30 µl) was carefully placed on the polymer film and the contact angle was recorded over time. The mean contact angle was calculated as average angle of 2 to 5 sec after the droplet was set on the surface. To investigate the advancing and receding contact angle, the drop volume was increased and decreased using a capillary tube [30], [31].

#### Cell Culture

The murine pre-osteoblast cell line MC3T3-E1 was obtained from tthe American Type Culture Collection (ATCC, #CRL-2593) and cultured in DMEM media (Gibco) supplemented with 10% fetal bovine serum (Hyclone) and kept in a humidified incubator at 37°C and 5% CO_2_. For biocompatibility experiments, cells were seeded at a density of 1.5×10^4^ cells per substrate (∼4.9 cm^2^) and cultured for 24 hours. After fixation with 4% paraformaldehyde, cells were stained with Phalloidin-AlexaFluor488 (Life Technologies) and 4′,6-Diamidin-2-phenylindol (DAPI, Life Technologies) to visualize actin filaments and the cell nucleus respectively. Experiments were done in duplicates and at least 60 cells of each condition were used for analysis. The cell form factor Φ was calculated as per the following equation:
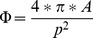
Where, A is the area of the cell and p the perimeter of a cell, using Image-J software (Version 1.47p, National Institutes of Health, USA). A Φ value of 1 would correspond to a cell with round morphology, and a Φ value of close to zero, would correspond to a highly elongated cell.

## Results and Discussion

### Monomer synthesis

**Figure 3:**
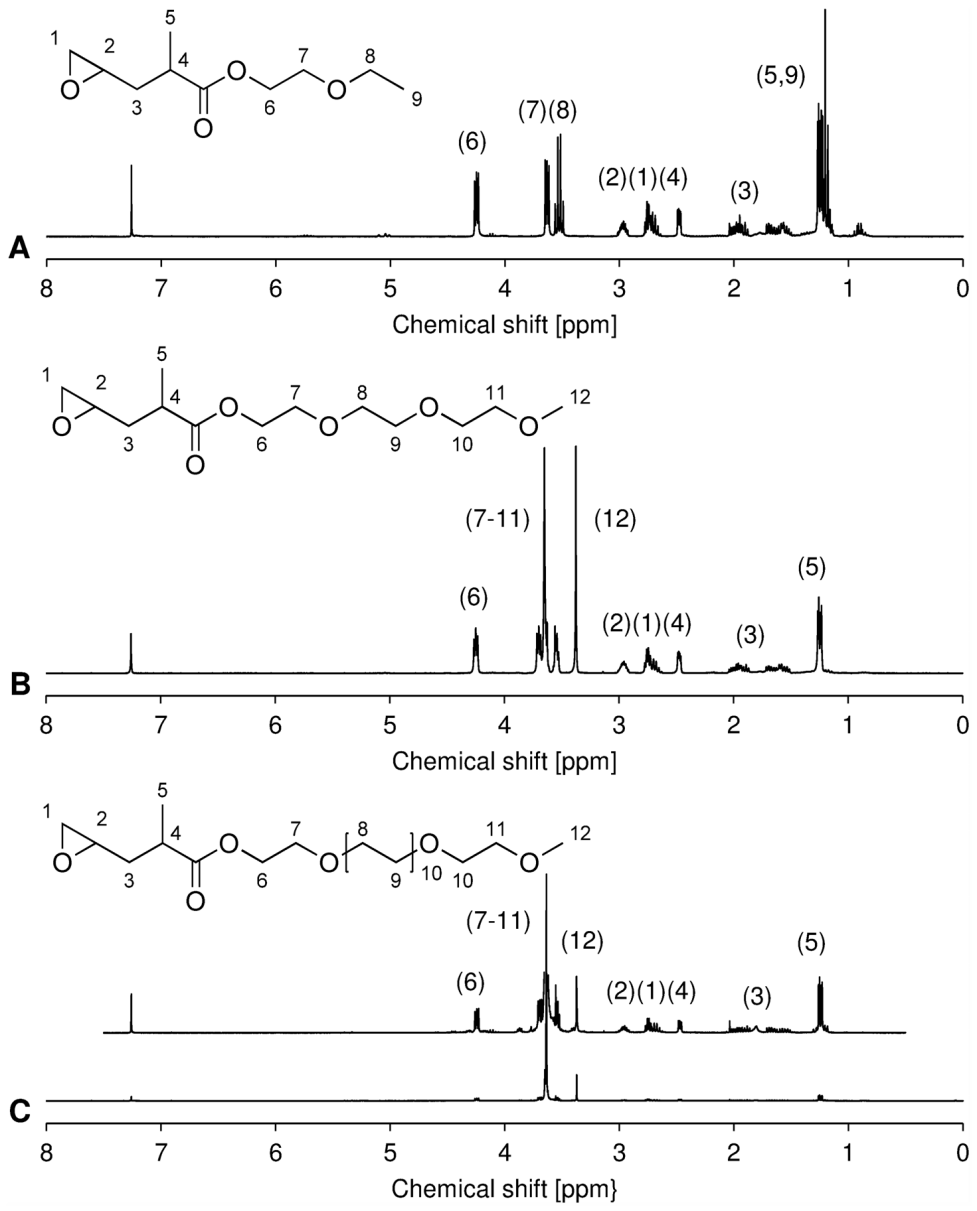
General reaction scheme of the copolymerization of CL and OEG-MPO: Shown here, as an example is the copolymerization of 3EG-MPO.

Three novel oligo(ethylene glycol) (OEG) functionalized epoxy esters, with OEG repeats of 1, 3, and 12 (1EG-MPO, 3EG-MPO, and 12EG-MPO, respectively) were synthesized in a two-step synthesis. The choice of the OEG moieties was limited by the commercial availability of these polyethers as monohydroxy derivatives in high purity. In the first step, OEG-MP ester was synthesized via Steglich esterification with the corresponding OEG, and in the subsequent step the terminal double bond in the OEG-MP ester was epoxidized using Oxone (**Figure 1**) [28]. The Steglich esterification was employed due to the mild reactions conditions that are well tolerated by oligo ethers and the mild basic workup conditions that afford high yields of the esters with almost no byproducts. As a result, the ester educt can be epoxidized with Oxone to the epoxy esters in high yields and obtained in high purity after flash chromatography. This is critical as monomer purity can significantly impact ROP in the presence of organometallic catalysts. The ester intermediates were obtained in 72, 68 and 58% yields, respectively for 1EG-MP, 3EG-MP and 12EG-MP. In the epoxidation step a tight control over the pH was critical to ensure that the OEG-MP ester did not hydrolyze. The epoxy esters were obtained in yields of 77%, 67%, and 62% in the case of 1EG-MPO, 3EG-MPO, and 12EG-MPO, respectively. In order to improve the overall yield, the unreacted unsaturated ester was recovered and reused. The purity of the monomer was established using ^1^H-NMR (**Figure 3**).

### Copolymerization

**Figure 4:**
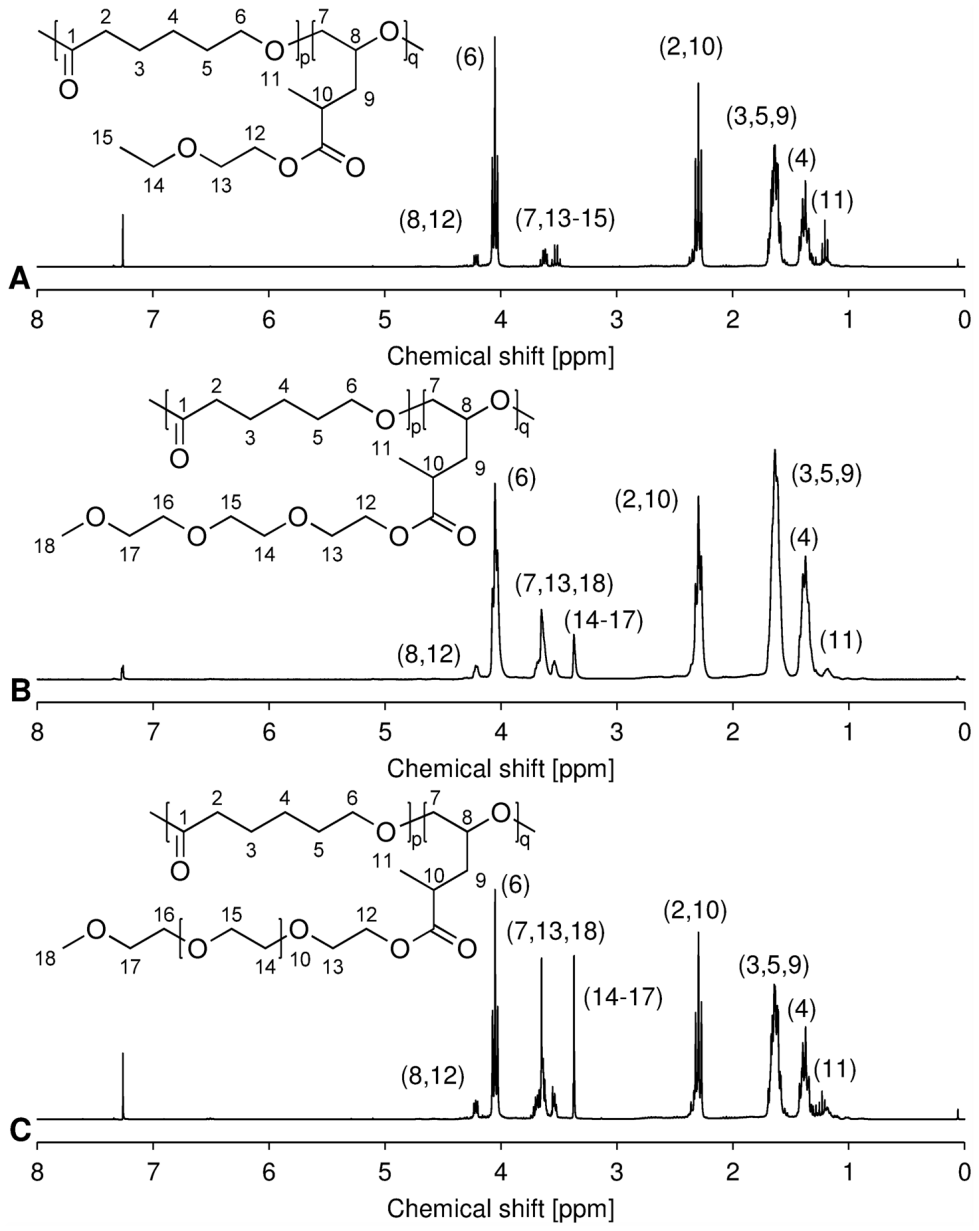
^1^H-NMR spectra of poly(CL-*co*-OEG-MPO). A poly(CL-*co*-1EG-MPO); B poly(CL-*co*-3EG-MPO); C poly(CL-*co*-12EG-MPO).

The copolymerization conditions were based on the optimization study undertaken earlier [27]. Based on these studies SnOct_2_∶BnOH initiator was selected and the ROP was carried out in bulk at 130°C for 48 h. The copolymerization compositions investigated in this study are shown in **Table 1** and they ranged from 5–20 mole percent of OEG-MPO in the feed. Typical proton NMR spectra of the copolymers are shown in **Figure 4**.

**Table 1:** Overview of the key properties of poly(CL-*co*-OEG-MPO) copolymers.

Entry	Copolymer	Feed ratio [CL∶epoxide]	Incorp. Ratio [CL∶epoxide]	Mn [kg/mol]	PDI	Yield [%]
a	Poly(CL-*co*-1EG-MPO)	95∶05	97.2∶2.8	6.43	1.83	54
b	Poly(CL-*co*-1EG-MPO)	90∶10	95.3∶4.7	4.86	1.67	51
c	Poly(CL-*co*-1EG-MPO)	85∶15	92.7∶7.3	4.00	1.49	51
d	Poly(CL-*co*-1EG-MPO)	80∶20	91.2∶8.8	3.67	1.51	32
e	Poly(CL-*co*-3EG-MPO)	95∶05	97.5∶2.5	7.74	1.70	49
f	Poly(CL-*co*-3EG-MPO)	90∶10	95.7∶4.3	4.36	1.54	47
g	Poly(CL-*co*-3EG-MPO)	85∶15	93.0∶7.0	4.06	1.38	26
h	Poly(CL-*co*-3EG-MPO)	80∶20	91.8∶8.2	3.81	1.49	23
i	Poly(CL-*co*-12EG-MPO)	95∶05	98.0∶2.0	15.62	2.13	44
j	Poly(CL-*co*-12EG-MPO)	90∶10	96.0∶4.0	3.78	1.64	29
k	Poly(CL-*co*-12EG-MPO)	85∶15	94.7∶5.3	3.01	1.33	16
l	Poly(CL-*co*-12EG-MPO)	80∶20	92.1∶7.9	1.23	1.92	14
m	Poly(ε-caprolactone)			49.5	1.51	93

MPO: 2-methyl-4-pentenoate oxide; 1EG-MPO: 2-ethoxyethanol-MPO; 3EG-MPO: trimethylene glycol monomethyl ether-MPO; 12EG-MPO: poly(ethylene glycol) monomethyl ether-MPO.

#### OEG-MPO incorporation efficiency and impact on molecular weight

**Figure 5:**
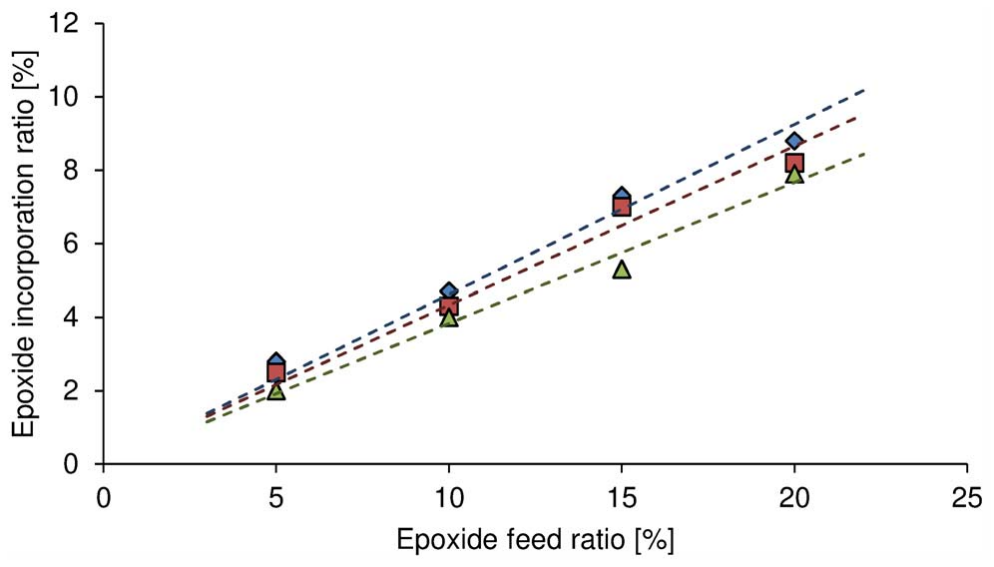
Incorporation efficiency of OEG-MPO in CL copolymers. Diamond: poly(CL-*co*-1EG-MPO); square: poly(CL-*co*-3EG-MPO); triangle: poly(CL-*co*-12E-MPO).

The incorporation of the epoxy ester comonomer in the copolymer was determined by ratiometric analysis of the signal associated with the OEG moieties and the dominant signals of the methylene protons associated with CL. The incorporation showed linear correlation to the comonomer ratio in the feed (**Figure 5**). In a given class of epoxide monomer, increasing the epoxide in the feed resulted in linear increase in incorporation. While the incorporation efficiency of 1EG-MPO and 3EG-MPO were comparable, i.e., 46% and 43%, and in agreement with earlier results where a 58% efficiency was observed for the ethyl ester of MPA [27], in the case of 12EG-MPO co-monomer however, a further 20% decrease in incorporation efficiency (38%) was observed. This suggests that steric effects imposed by the long OEG moiety, in combination with the overall lower reactivity of MPO esters, are the limiting factors in achieving higher comonomer content. In general, one can conclude that the for a given polymer composition increasing OEG chain length resulted in a decrease in polymer yield (**Table 1**), as for example, in the case of poly(Cl-*co*-OEG-MPO) 90∶10, the yield was almost halved when the OEG moiety was 12EG. This is again consistent with an overall diminution in copolymerization propensity due to decreased reactivity of the OEG-MPO monomers and their low capacity for self-polymerization. Another factor might be the side reactions associated with the epoxide and the EG moieties during copolymerization [23], [32]. Notwithstanding, a reasonably high incorporation of around 8% was achieved with a 20% ratio of the epoxide in the feed. The OEG chain length and feed ratio also impacted the molecular weight of the copolymers. While a general trend towards lower molecular masses was observed with increasing epoxy comonomer ratio in the feed, this appeared to have no impact on the polydispersity (PDI) of the copolymers, with an exception of poly(CL-*co*-12EG-MPO), 95∶5, where molecular masses in excess of 15,000 g/mol were obtained but at the expense of a significant increase in PDI. A possible explanation for this anomaly could be the low incorporation of 12EG-MPO in the copolymer that then favors addition of CL monomer units over the epoxide, as this is consistent with the rather drastic reduction in molecular mass observed in the case of 12EG-MPO with increasing feed ratios. The emergence of different copolymer species may be ruled out as the size exclusion traces showed a unimodal distribution.

#### Thermal behavior of copolymers

The thermal properties of the copolymers are summarized in **Table 2**. The glass transition temperatures of all copolymers increased with increasing epoxide content in the copolymer, with a maximum value of −13°C observed in poly(Cl-*co*-12EG-MPO) of 92∶8 composition. This trend is more pronounced within a monomer group with increasing mole %, but also present albeit to a lesser extent when comparing copolymers of similar composition (Table 2, entries c and g) across all three monomers. We had established in the copolymerization study with ethyl ester of MPO that the low reactivity of the epoxy esters result in a statistically random copolymer [27]. This would imply that the flexible moieties in the polymer backbone that originate in the epoxy ester comonomer are randomly distributed along the copolymer backbone and the crystalline domains are derived from the CL regions and this is consistent with the appearance of a single T_g_. This also explains in part the dramatic reduction in T_g_ that is observed. The absence of a T_g_ in some instances is however not easily explained and need further study. The overall trend is nevertheless consistent with the prediction by the Fox equation [33], [34].

**Table 2:** Summary of the thermal properties of poly(CL-*co*-OEG-MPO) copolymers.

Entry	Copolymer	Incorp. Ratio [CL∶epoxide]	Tg [°C]	Tm1 [°C]	Tm2 [°C}
a	Poly(CL-*co*-1EG-MPO)	97.2∶2.8	−41	48	50
b	Poly(CL-*co*-1EG-MPO)	95.3∶4.7	−36	43	49
c	Poly(CL-*co*-1EG-MPO)	92.7∶7.3	−31	38	45
d	Poly(CL-*co*-1EG-MPO)	91.2∶8.8	−16	36	44
e	Poly(CL-*co*-3EG-MPO)	97.5∶2.5	−39	47	49
f	Poly(CL-*co*-3EG-MPO)	95.7∶4.3	−38	40	47
g	Poly(CL-*co*-3EG-MPO)	93.0∶7.0	−21	36	43
h	Poly(CL-*co*-3EG-MPO)	91.8∶8.2	-	35	42
i	Poly(CL-*co*-12EG-MPO)	98.0∶2.0	−36	48	52
j	Poly(CL-*co*-12EG-MPO)	96.0∶4.0	-	45	48
k	Poly(CL-*co*-12EG-MPO)	94.7∶5.3	−13	39	45
l	Poly(CL-*co*-12EG-MPO)	92.1∶7.9	-	34	41
m	Poly(ε-caprolactone)		−61		63

MPO: 2-methyl-4-pentenoate oxide; 1EG-MPO: 2-ethoxyethanol-MPO; 3EG-MPO: trimethylene glycol monomethyl ether-MPO; 12EG-MPO: poly(ethylene glycol) monomethyl ether-MPO.

**Figure 6:**
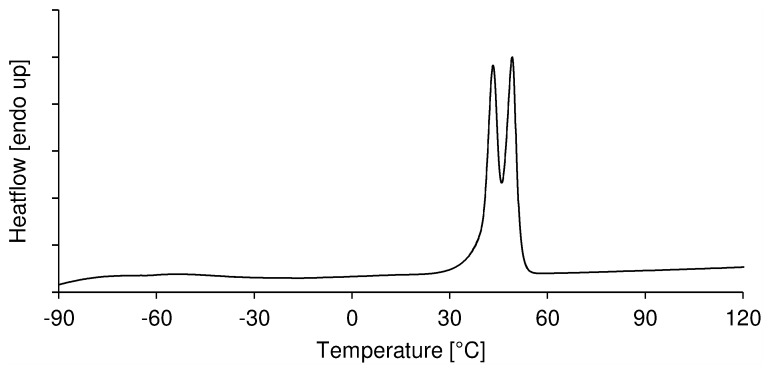
DSC thermogram of poly(CL-*co*-1EG-MPO) with CL∶1EG-MPO = 95.3∶4.7. Melting peaks at 43°C and 49°C.

A typical DSC thermogram of poly(CL-*co*-OEG-MPO) is shown in **Figure 6**. In comparison to pure PCL the copolymers exhibit two sharp overlapping melting transitions (T_m1_ and T_m2_), which are lower than pure PCL (**Table 2**), indicating the presence of two crystalline regimes in the copolymers. Both the melting transitions showed an inverse relationship to increasing OEG content in the copolymer, with the lower of the two melting transitions showing a more distinct change than the upper melting temperature. The fact that both of these melting transitions are significantly lower than that of pure PCL suggests that the both crystalline domains are composed of CL and OEG moieties and might be indicative of a complex phase segregation that needs to be further investigated. Such a change in crystallization behavior was not observed in the poly(CL-*co*-EMPO) system that was reported recently [27].

### Surface characterization of copolymer films

The films of copolymers were analyzed using water contact angle and atomic force microscopy to discern the effects of OEG incorporation on wettability and surface topography.

#### Water contact angle analysis

**Figure 7:**
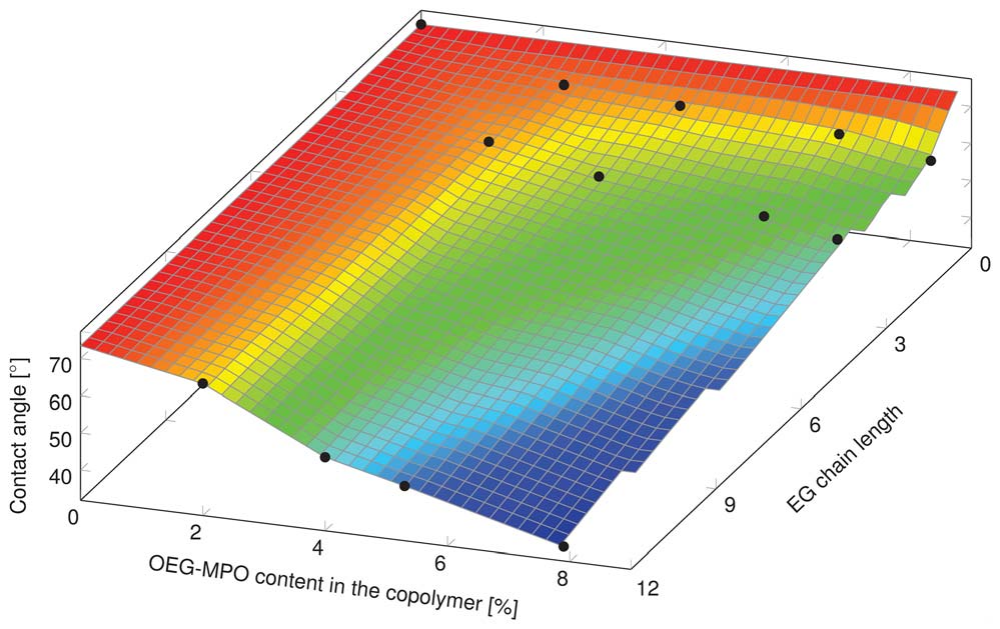
Static water contact angle of poly(CL-*co*-OEG-MPO) copolymers.

**Figure 8:**
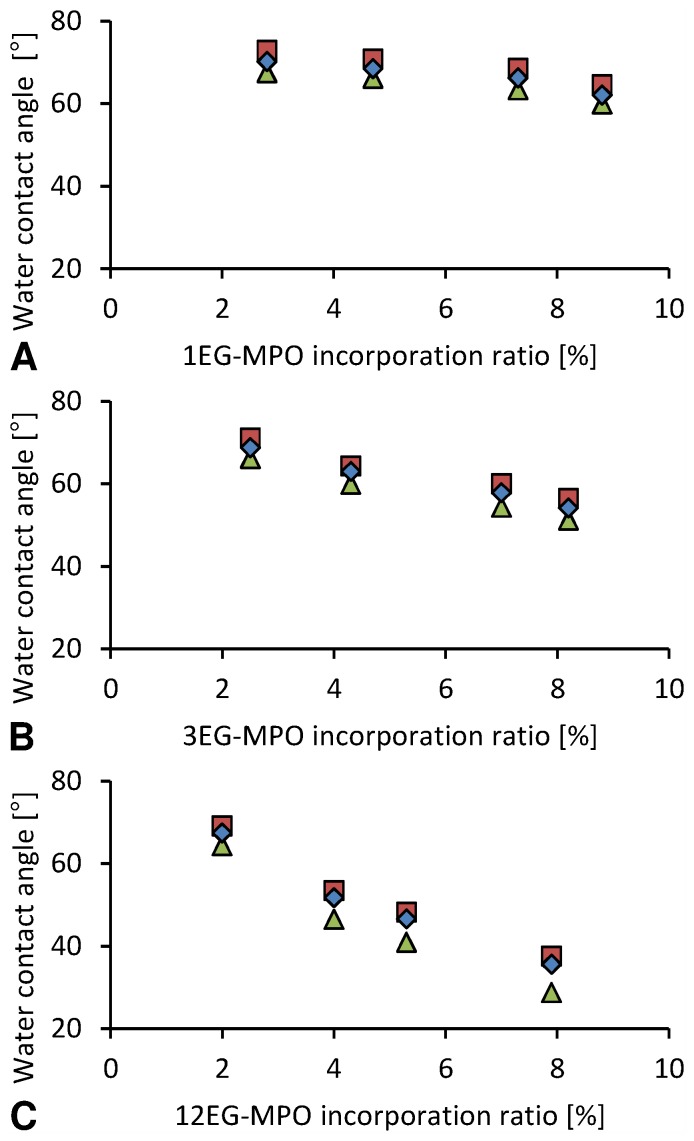
Advancing and receding contact angle (CA). Diamond: static CA; square: advancing CA; triangle: receding CA.

The dependency of static water contact angle (CA) on OEG is summarized in **Figure 7**. The change in CA as function of OEG mole percent (°/%) in the co-polymer scaled with OEG chain length with a 1.2°/%, 2.3°/%, and 5.0°/% change, for 1EG-MPO, 3EG-MPO and 12EG-MPO, respectively. The lowest contact angle in the copolymer systems with EG chain lengths of 1, 3 and 12 was 62°, 54° and 36°, respectively and was obtained at an OEG co-monomer mole ratio of around 8%. Not surprisingly, the rate at which the surface wettability decreased in relation to the co-monomer content was the steepest in the case of 12EG-MPO, which is essentially equivalent to a PEG moiety of 550 g/mol. However, this dramatic reduction in wettability comes at the expense of surface homogeneity, which was assessed by measuring the advancing (aCA) and receding (rCA) water contact angles (**Figure 8**). The deviation of the aCA and rCA from the static CA can provide some insights into the surface compositional homogeneity, mobility of polymer chains at the surface and surface perturbations. In accordance with the chemical structure of the OEG moieties, copolymers composed of shorter 1EG and 3EG elements showed no significant hysteresis whereas, copolymers composed of 12EG elements showed a divergence from static values with increasing epoxide content. This is expected as the 12EG moiety will have more mobility at the polymer-water interface in comparison to 1EG and 3EG and as such while increasing the affinity of the surface to water molecules will also render the surface more dynamic. These observations clearly demonstrate that both the OEG chain length and content are important variables in controlling the surface hydrophilicity.

#### Roughness of copolymer films

**Figure 9:**
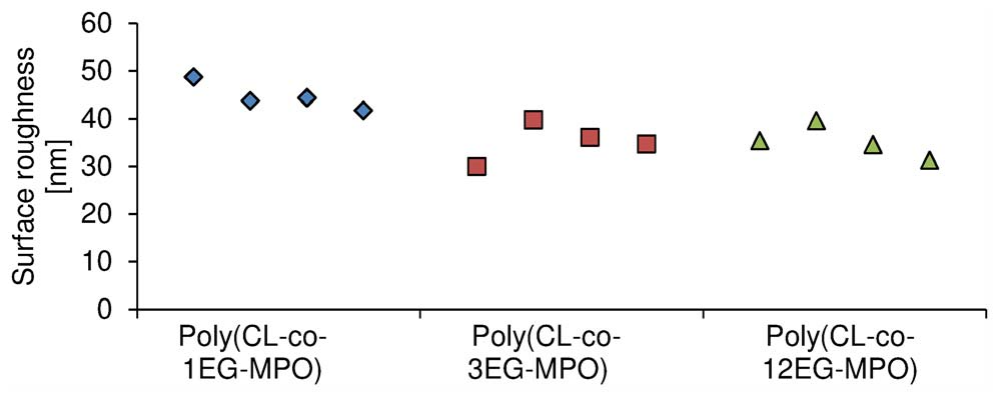
Surface roughness of copolymer films as determined using atomic force microscopy. Roughness is reported as root mean square roughness (Rq).

Since surface roughness can impact wettability the copolymer films were imaged using tapping mode AFM and the root mean square roughness (R_q_) values as a function of copolymer composition is presented in **Figure 9**. The Rq of copolymer film surfaces ranged from 31 nm to 48 nm depending on the copolymer composition. However, increasing EG lengths (3 and 12) resulted is a modest reduction of Rq, to an average of 35 nm in comparison to 1EG-MPO copolymer films which were in the range of 44 nm. The absence of any significant differences in surface morphology and Rq allows one to conclude that the observed changes in both static and dynamic CAs are most likely due to chemical variables and the segregation of OEG moieties to the film surface upon wetting rather than physical variables.

### MC-3T3-E1 morphology on hydrophilic PCL-copolymer films

**Figure 10:**
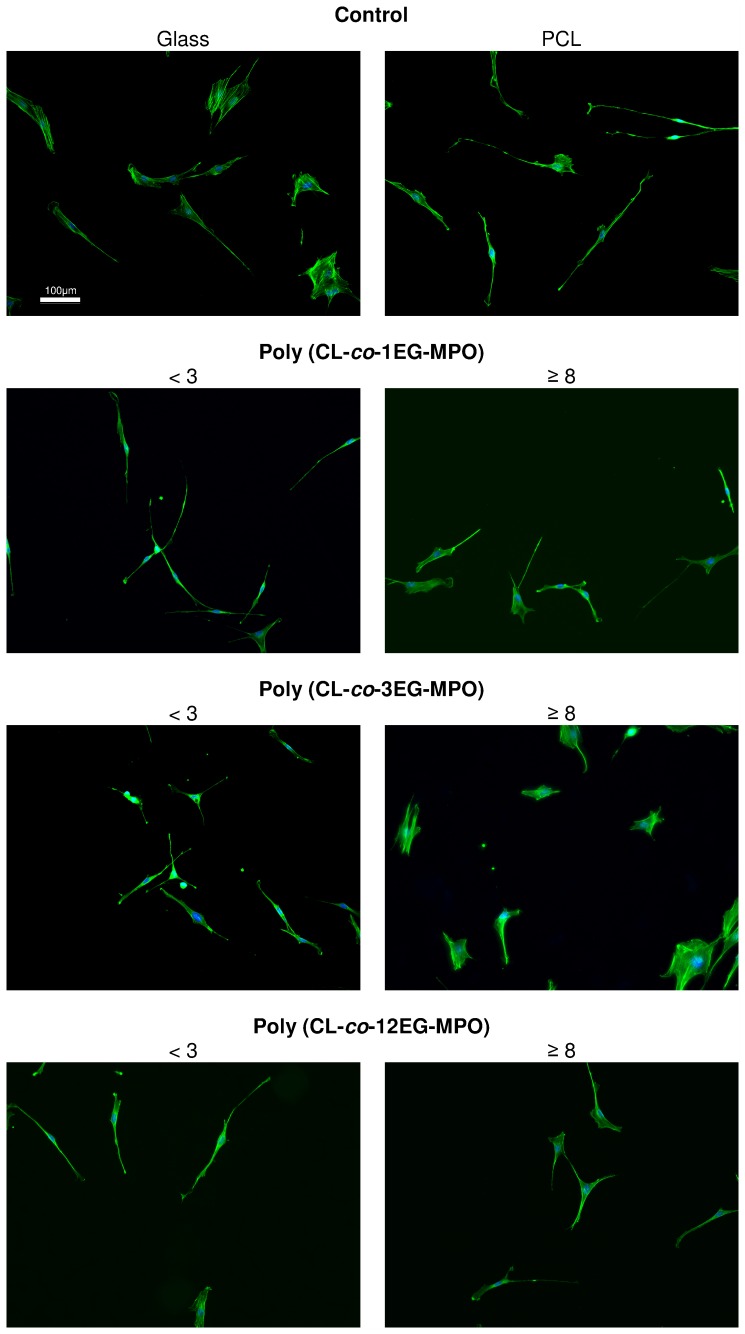
Fluorescence images of MC3T3-E1 cells on poly(CL-*co*-OEG-MPO) copolymer films. Green: F-actin stress fibers; blue: nucleus. Note the change in cell morphology from a highly anisotropic cell morphology on unmodified PCL and low oligo-EG-PCL surfaces to a more isotropic (rounded) morphology on poly(CL-co-3EG-MPO) with 8% incorporation.

**Figure 11:**
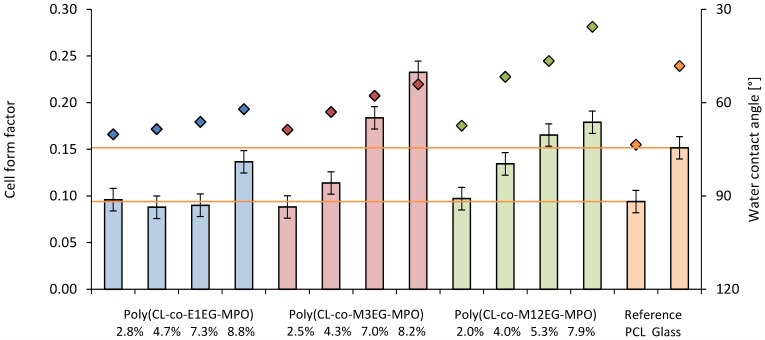
Influence of the poly(CL-*co*-OEG-MPO) polymer film substrate and water contact angle on cell shape. Bars: Cell form factor; dots: Water contact angle.

**Figure 12:**
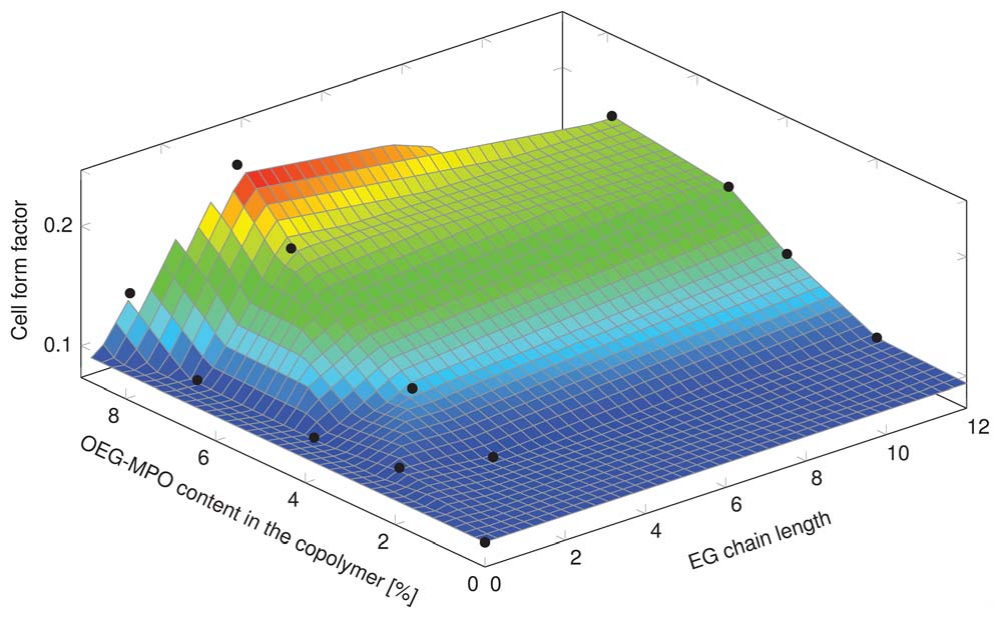
Relationship between MC-3T3-E1 shape factor (Φ), OEG chain length and incorporation.

Since one of the intended applications of this co-polymer is the coating of devices a preliminary study was undertaken with murine pre-osteoblasts MC-3T3-E1. Since cell shape is coupled to cell function, the impact of changes in wettability imparted by the introduction of OEG units, on cell shape was investigated. The changes to cell shape were quantified by determining the cell shape factor (Φ). A Φ value of 1 corresponds to a cell with a round morphology and a value close to zero is indicative of a highly elongated morphology. As anticipated, the incorporation of hydrophilic OEG moieties influenced the morphology of the MC3T3-E1 cells on the copolymer substrates (**Figure 10**). While cells on unmodified PCL surfaces exhibited highly elongated morphology, on co-polymer surfaces with greater than 8 mole-% OEG-MPO content, a more rounded morphology was observed. This change in morphology was comparable to what is observed on glass substrates. This is borne out by the cell form factor analysis (**Figure 11**) and the observed correlation between OEG content, wettability and Φ values. An important finding of this study is the presence of an optimum in both OEG chain length and copolymer composition with regards to cell morphology (**Figure 12**). This optimum was identified to occur at medium length OEG side chains (3 EG units) with incorporation of seven percent and greater. However, longer OEG units approaching short PEG chains (i.e., 12EG-MPO) actually had no pronounced effect on Φ. This is a counter intuitive finding that has implications for the design of PCL copolymers with improved cell-material interactions. We theorize that longer OEG such as 12EG-MPO in fact reduce the surface density of hydrophilic molecules that is essential to encourage uniform adsorption of serum proteins with minimum denaturation. The observation that 3EG-MPO at incorporation higher than 7% promote the most significant change in Φ, is consistent with the argument presented above, as with higher backbone incorporation, the statistical distribution of hydrophilic regions would increase. We further theorize that these observations are due to the OEG side chains of the copolymer that are present at the film surface and therefore can directly interact with the cells. This is consistent with both static and dynamic contact angle data (**Figure 8**) where 12EG-MPO surfaces show the highest hysteresis (4.5 to 9.0) and changes in wettability (67.5° to 35.5°), suggesting the presence of a more heterogeneous surface in comparison to 3EG-MPO. 3EG-MPO copolymer surfaces on the other hand show the lowest hysteresis (5.0 to 5.5), while still showing increasing wettability (68.5° to 54.0°). However, establishing an empirical relationship between polymer backbone chemistry and cell shape is not possible as shape is mediated by many variables like protein adsorption, conformational changes in adsorbed proteins, topography, and rigidity of cell-contact points among other all of which may be altered by changes to surface hydrophilicity.

## Conclusions

We have introduced a novel class of ε-caprolactone based copolymers that present OEG derivatives along the backbone linked via degradable ester linkages. Under standard bulk ROP using SnOct_2_∶BnOH as initiating system, poly(CL-*co*-OEG-MPO) copolymers with molecular masses of up to 15,000 g/mol were successfully synthesized with low PDI. The functional group density in the copolymer backbone showed a linear correlation with the epoxide content in the copolymerization feed, thereby enabling the synthesis of copolymers with up to 8% by monomer mole ratio. The static water contact angles of these OEG modified PCL copolymers showed an inverse correlation with increasing OEG content. This allows for a defined control of the hydrophilic characteristics of the PCL copolymer. Increasing the ethylene glycol chain length resulted in significant hysteresis in contact angle implying that the copolymer surface was dynamic. The data at hand suggests that poly(CL-*co*-OEG-MPO) improve cell spreading and possess a favorable profile for cell contacting applications. However, further studies need to be undertaken to fully ascertain the potential of this new family of modified CL copolymers in biomedicine. The hydrophilic PCL copolymers might be suitable for implantable devices and injectable systems for controlled release and as coatings for metallic stents.
